# Antarctic soil microbiomes encode structurally conserved and phylogenetically diverse beta‐lactamases

**DOI:** 10.1002/imo2.70118

**Published:** 2026-07-04

**Authors:** José Coche‐Miranda, Patricio Arros, Nicolás Canales, Camilo Berríos‐Pastén, Gastón Azziz, Rosalba Lagos, Julieta Orlando, Francisco P. Chávez, Andrés E. Marcoleta

**Affiliations:** ^1^ Grupo de Microbiología Integrativa, Laboratorio de Biología Estructural y Molecular BEM, Departamento de Biología, Facultad de Ciencias Universidad de Chile Santiago Chile; ^2^ Microbiology Laboratory, Faculty of Agronomy Universidad de la República Montevideo Uruguay; ^3^ Molecular Microbiology Unit Clemente Estable Biological Research Institute Montevideo Uruguay; ^4^ Laboratorio de Ecología Microbiana, Departamento de Ecología, Facultad de Ciencias Universidad de Chile Santiago Chile; ^5^ Instituto Milenio Biodiversidad de Ecosistemas Antárticos y Subantárticos Santiago Chile; ^6^ Laboratorio de Microbiología de Sistemas, Departamento de Biología, Facultad de Ciencias Universidad de Chile Santiago Chile

**Keywords:** antibiotic inactivation, beta‐lactamase, cryosphere, natural resistome, structure‐guided metagenomics

## Abstract

Antimicrobial resistance is a major global health threat shaped not only by clinical settings but also by environmental reservoirs, in line with the One Health framework. Antarctic and Subantarctic soils represent a largely unexplored component of the environmental resistome, particularly beta‐lactamases, whose diversity, structural features, and biogeographic patterns remain poorly resolved. Here, we applied an integrative metagenomic approach combining sequence‐, structure‐, and function‐based similarity to systematically identify putative beta‐lactamase orthologs across an extensive collection of Antarctic and Subantarctic soil metagenomes. We detected multiple beta‐lactamase classes and families, with a predominance of class A serine beta‐lactamases and subclass B3 metallo‐beta‐lactamases. Several previously uncharacterized families and divergent variants were identified, many of which retain conserved catalytic architectures. Beta‐lactamase diversity and relative abundance were strongly patterned across three major biogeographic clusters: Subantarctic islands, the Antarctic Peninsula and surrounding islands, and continental cold deserts, mirroring shifts in microbial community composition. The Antarctic Peninsula and maritime Antarctica exhibited the highest overall beta‐lactamase abundance. Analysis of more than 3500 metagenome‐assembled genomes indicated that most beta‐lactamases were encoded by members of *p_Pseudomonadota* and *p_Bacteroidota*, with additional representatives distributed across other bacterial phyla. A subset was linked to mobile genetic elements, suggesting a latent but limited potential for horizontal gene transfer. Together, these findings reveal a geographically organized and compositionally diverse repertoire of beta‐lactamases in (sub)Antarctic soils, underscoring the evolutionary persistence of conserved catalytic frameworks in cold environments and highlighting the contribution of remote ecosystems to global resistome diversity.

## Highlights

- Antarctic soils harbor a diverse and geographically structured reservoir of beta‐lactamases.
- Class A and subclass B3 beta‐lactamases dominate across (sub)Antarctic metagenomes.
- Integrative sequence‐structure‐function analysis reveals novel and highly divergent beta‐lactamases.
- Beta‐lactamases are primarily encoded by *p_Pseudomonadota* and *p_Bacteroidota* across Antarctic soils.

## Graphical abstract

An integrative metagenomic framework combining sequence, structural, and functional inference reveals a phylogenetically diverse and structurally conserved repertoire of putative beta‐lactamases across Antarctic soil microbiomes, with predominance of class A and subclass B3 enzymes and limited but detectable associations with mobile genetic elements.

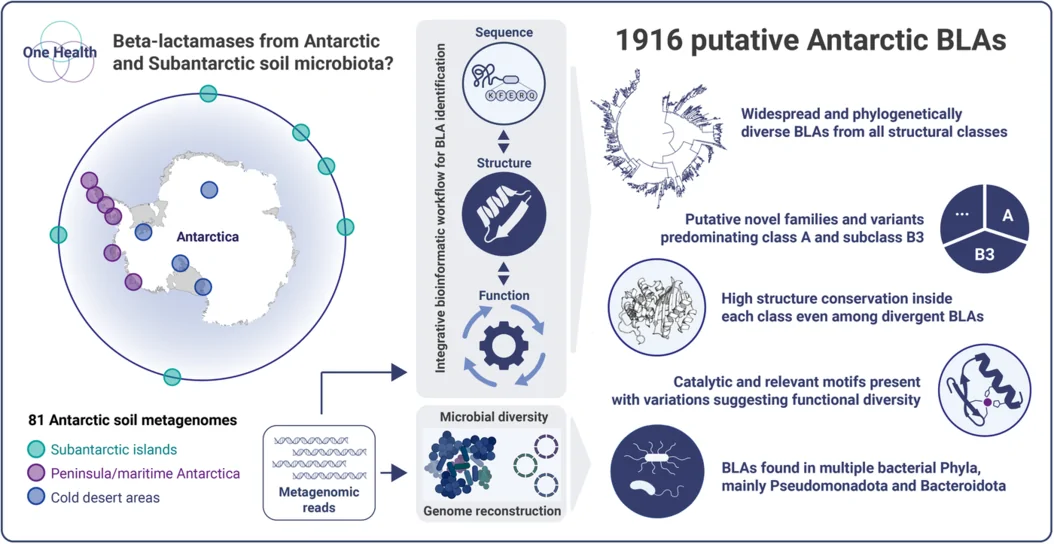

## 1 INTRODUCTION

Antimicrobial resistance (AMR) is a major global health challenge, responsible for an estimated 1.3 million deaths annually and projected to cause up to 10 million deaths per year by 2050 if effective countermeasures are not implemented [1]. Addressing AMR requires understanding not only clinical dynamics but also its environmental origins, in line with the One Health framework, which integrates the interconnected roles of humans, animals, and ecosystems [2]. Within this context, it has been widely demonstrated that the antibiotic resistance genes (ARGs) are ancient and ubiquitous, and that environmental microbiota, even in remote and extreme environments, constitute reservoirs from which resistance determinants may emerge [3, 4, 5].

Among ARGs of particular concern are those encoding beta‐lactamases (BLAs), the most widespread and efficient resistance mechanism against beta‐lactam antibiotics in Gram‐negative bacteria [6, 7, 8]. Beta‐lactams remain the most extensively used antimicrobial agent in clinical practice [9], underscoring the importance of understanding the diversity and evolution of BLAs. Based on sequence similarity and structural features, BLAs are classified into two major groups: serine beta‐lactamases (SBLs; classes A, C, and D) and metallo‐beta‐lactamases (MBLs; subclasses B1, B2, and B3) [10, 11]. SBLs belong to the PBP‐like superfamily and share conserved active‐site motifs (SXXK, SXN, and KTG(T/S)), whereas MBLs belong to the MBL‐fold superfamily and coordinate zinc ions through conserved histidine and aspartate residues [12, 13]. MBLs are characterized by an alpha‐beta‐beta‐alpha‐fold, and two conserved active‐site metal‐binding motifs involving H116/H118/H196 and D120/H121/H263, along with D221 that coordinates both metals. Both groups of beta‐lactamases are evolutionarily ancient, with origins estimated at approximately 2–2.5 billion years ago [12, 14], suggesting that their primary biological roles extend beyond clinical antibiotic resistance.

BLAs have been identified across diverse environments, including ecosystems with minimal human impact [15, 16]. Maritime Antarctic soils, in particular, have been reported to harbor potential ARGs, including BLAs, and to host bacteria resistant to multiple beta‐lactam antibiotics [5, 17, 18]. Nevertheless, the distribution, taxonomic origin, and structural characteristics of beta‐lactamases across Antarctic soil environments remain incompletely understood.

To date, only a limited number of studies have explored BLAs in Antarctic metagenomes. A survey of 11 microbial mat metagenomes along a latitudinal gradient identified putative BLAs from classes A–D, with class C predominating [19]. However, soils, expected to exhibit higher microbial and genetic diversity than microbial mats [20, 21], have not been comprehensively surveyed across distinct Antarctic regions. Moreover, previous studies did not systematically assess the taxonomic distribution, structural conservation, or evolutionary placement of the identified BLA sequences, limiting insight into their ecological roles and evolutionary trajectories.

Identifying beta‐lactamase orthologs based solely on sequence identity presents important limitations, particularly for deeply divergent environmental variants. Remote homologs may exhibit low similarity to clinically characterized enzymes, increasing the likelihood of both false positives and false negatives in conventional searches. We therefore hypothesized that Antarctic soils encode a broad and largely unexplored repertoire of beta‐lactamases that can be more reliably detected by integrating multiple complementary layers of information derived from metagenomic data.

To test this hypothesis, we applied an integrative metagenomic workflow combining sequence similarity, structural prediction, and multidimensional functional inference to an extensive collection of soil metagenomes from Subantarctic islands, maritime Antarctica, and continental cold desert environments. This strategy enabled systematic identification of putative beta‐lactamase orthologs while reducing misclassification within large protein superfamilies, thereby allowing a comprehensive assessment of their diversity, structural organization, and distribution across Antarctic ecosystems.

## 2 RESULTS

### Biogeographic patterns of microbial community structure across Antarctic soils

We constructed a set of 81 soil metagenomes, including six from sub‐Antarctic Islands (SI): South Georgia, Marion, Kerguelen, Bartolome, Possession, and Bouvet Islands. Additionally, 37 metagenomes from the Antarctic Peninsula and surrounding islands (AP), mainly the South Shetland Archipelago; and 38 from high‐latitude cold desert areas (CD), primarily the McMurdo Dry Valleys, the Shackleton Glacier, and the Union Glacier (Figure 1A). The complete list of metagenomes included in this study, along with their accession numbers and relevant metadata, is provided in Table S1.

**Figure 1:**
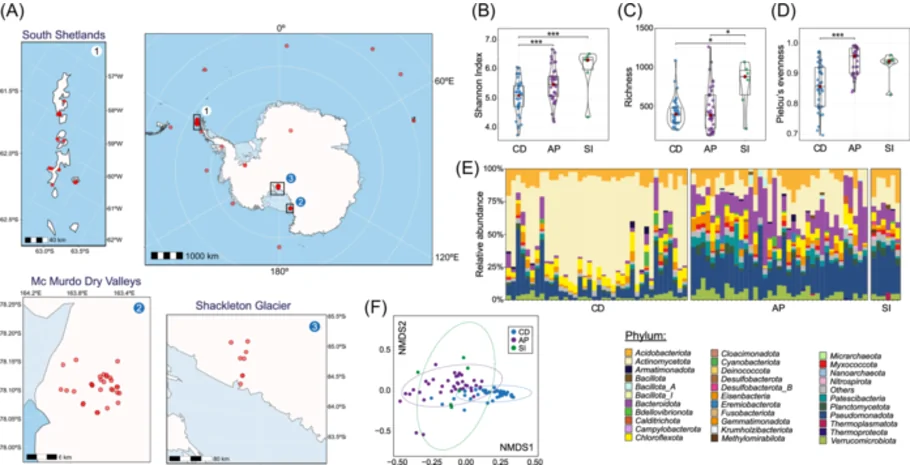
Geographic distribution and microbial diversity of Antarctic and Subantarctic soil metagenomes. (A) Map showing the geographic location of the 81 soil metagenomes included in this study. Insets 1–3 show enlarged views of the regions with higher sample density. (B–D) distribution of diversity indices across cold desert (CD), Antarctic Peninsula and surrounding islands (AP), and Subantarctic Islands (SI) soils, based on 43 essential single‐copy marker genes. *p_FDR‐adjusted_ < 0.05 and ***p_FDR‐adjusted_ < 0.001. (E) Relative abundance of microbial phyla across the analyzed metagenomes. (F) Non‐metric multidimensional scaling (NMDS) ordination based on Bray–Curtis dissimilarities calculated from family‐level taxonomic profiles. PERMANOVA indicated significant differences in microbial community structure between AP and CD and between CD and SI soils. Complementary ordination quality and beta‐dispersion analyses are shown in Figure S1.

First, we aimed to explore the diversity and taxonomic composition of the microbial communities inhabiting the different Antarctic soils studied. We used the SingleM pipeline, which is specially designed for microbial diversity analyses from shotgun metagenomic data based on a collection of 59 essential single‐copy genes (ESCGs) [22]. However, we only kept the results for 43 ESCGs because 16 were not observed in at least one sample. Shannon Index values differed significantly among soil types, with the lowest diversity in CD (median = 5.12), followed by AP (median = 5.55), and the highest SI (median = 6.40) (Figure 1B). This pattern likely reflects similar richness in CD and AP (median = 393 and 381, respectively), both markedly lower than in SI (median = 882) (Figure 1C). Additionally, evenness was high in AP and SI (medians: 0.95 and 0.94) but significantly reduced in CD (median: 0.86) (Figure 1D).

Regarding microbial community structure, the SI and AP soils showed a more similar phyla composition, predominating *p_Pseudomonadota* (mainly *o_Burkholderiales*), *p_Bacteroidota* (mainly *o_Chitinophagales*), *p_Acidobacteriota* (mainly *o_Pyrinomonadales*), and, to a lesser extent, *p_Actinomycetota* (mainly *o_Acidimicrobiales*) (Figure 1E). Conversely, in most CD soils, *p_Actinomycetota* (mainly *o_Solirubrobacterales*) predominated, whereas *p_Bacteroidota*, *p_Pseudomonadota*, *p_Gemmatimonadota*, and *p_Patescibacteria* were markedly less abundant. In agreement, beta‐diversity dissimilarity analysis at the family level revealed a more similar taxonomic composition between SI and AP soils, whereas the CD soils clustered apart, due to their distinct community structure (Figure 1F, Figure S1).

### An integrative strategy for Antarctic beta‐lactamase identification

To identify beta‐lactamase orthologs, we applied an integrative workflow that combines complementary protein assembly and clustering strategies with sequence‐, structure‐, and function‐based similarity embeddings to generate confident protein ortholog clusters (Figure 2). This approach enables discrimination between true beta‐lactamase orthologs and phylogenetically related proteins with different functions.

**Figure 2:**
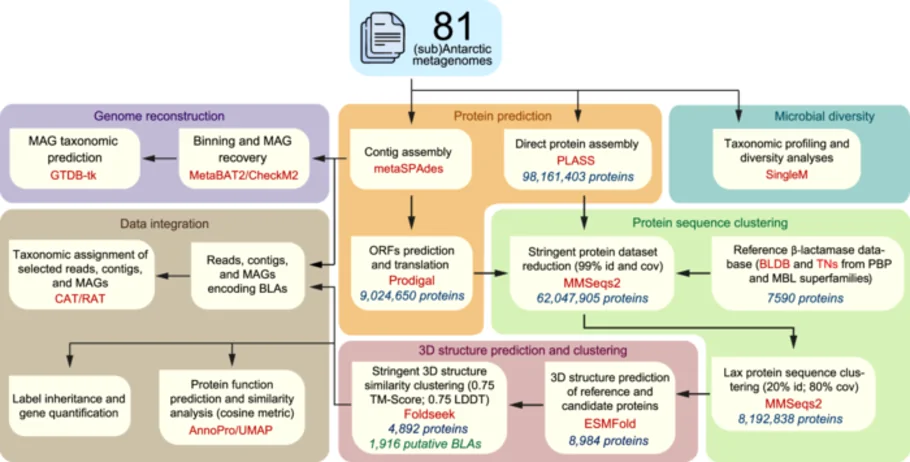
Integrative bioinformatic workflow for Antarctic beta‐lactamase identification and microbial diversity analysis. The workflow summarizes the main analytical stages used in this study, including metagenome assembly, direct protein assembly, protein prediction, protein sequence clustering, structure prediction and structural clustering, functional similarity analysis, genome reconstruction, taxonomic assignment, gene quantification, and microbial diversity profiling. Main workflow modules are highlighted by color. Bioinformatic tools used in each step are indicated in red, intermediate protein counts are shown in blue, and the final number of putative Antarctic beta‐lactamases is shown in green.

Metagenomic reads from the 81 soil datasets were assembled at the contig level using metaSPAdes, followed by open reading frame (ORF) prediction, yielding approximately 9 million protein sequences. Additionally, direct protein assembly from metagenomic reads was performed using PLASS [23]. PLASS enables ultra‐fast, scalable protein‐level assembly, overcoming biases in contig reconstruction to uncover low‐abundance proteins, protein variants, and novel sequences that are often missed by traditional workflows. This complementary strategy increased the total number of predicted proteins to approximately 98 million.

To reduce redundancy, all predicted proteins were clustered at 99% sequence identity and 99% bidirectional coverage, resulting in approximately 62 million non‐redundant sequences. These were combined with curated reference beta‐lactamases from the Beta‐Lactamase DataBase (BLDB; 5,458 non‐redundant representatives) and a set of 24 phylogenetically related non‐beta‐lactamase proteins from the PBP‐like and MBL‐fold superfamilies used as negative controls (true negatives; TNs) (Table S2).

An initial clustering step at 20% sequence identity and 80% bidirectional coverage identified 3,502 Antarctic protein sequences grouping either with reference beta‐lactamases (2181 sequences) or with negative controls (1321 sequences). To further refine candidate selection, three‐dimensional structures were predicted for these Antarctic proteins, as well as for all reference beta‐lactamases and negative controls. Structure‐based clustering was then performed using strict similarity criteria (LDDT ≥ 0.75; TM‐score ≥ 0.75), enabling discrimination of beta‐lactamase orthologs from related proteins with distinct functions.

This integrative filtering yielded a final set of 1916 non‐redundant Antarctic beta‐lactamase candidates forming 25 clusters with reference beta‐lactamases (Figure 2; Table S3), and 692 proteins clustering with TNs. No mixed clusters containing both reference beta‐lactamases and negative controls were observed.

### Benchmarking and validation of beta‐lactamase identification

To evaluate the performance of our integrative approach, we compared the resulting candidate set with two widely used sequence‐based methods for beta‐lactamase detection: MMSeqs2, based on local sequence alignments, and HMMER, using hidden Markov models (HMMs) derived from beta‐lactamase profiles. Both methods were applied to the complete set of predicted proteins from the 81 Antarctic soil metagenomes (see Methods for details).

Using thresholds optimized through Receiver Operating Characteristic (ROC) analyses (Figure S2), MMSeqs2 and HMMER each detected the full set of 1916 structurally supported Antarctic beta‐lactamase candidates. At their respective optimal discrimination thresholds (defined by maximum Matthews Correlation Coefficient), MMSeqs2 identified 76,837 additional potential beta‐lactamase hits, whereas HMMER identified 80,186.

The union of both sequence‐based candidate sets comprised 92,283 non‐redundant proteins. When aligned against our structurally curated reference set, including confirmed beta‐lactamases and phylogenetically related non‐beta‐lactamase proteins, 52,348 (56.73%) were classified as putative beta‐lactamases, whereas 39,935 (43.27%) grouped with non‐beta‐lactamase homologs. This proportion indicates that reliance on sequence similarity alone would lead to substantial misclassification of deeply divergent homologs within large protein superfamilies.

Notably, 90.55% of these additional candidates exhibited amino acid identity values below 40% relative to reference beta‐lactamases (Figure S2). Within this low‐identity range, extensive overlap was observed among proteins from the PBP‐like superfamily, including carboxylesterase EstU1‐like proteins and class C beta‐lactamases. This convergence likely reflects shared evolutionary ancestry and highlights the reduced discriminatory resolution of sequence similarity‐based methods when evaluating deeply divergent putative orthologs.

In contrast, the integrative workflow incorporating structural similarity constraints yielded a more conservative set of 1916 non‐redundant beta‐lactamase candidates, all supported by coherent structural clustering. These findings indicate that combining structural and sequence information refines candidate selection, particularly within evolutionary distance ranges where sequence similarity alone provides limited discrimination.

Furthermore, most additional sequence‐based candidates belonged to the PBP‐like superfamily (79.79%), including carboxylesterase EstU1‐like proteins (29.77%), class C beta‐lactamases (26.82%), and class A beta‐lactamases (15.83%). Within the <40% identity range, these protein groups occupy overlapping regions of sequence similarity space, consistent with their shared evolutionary origin. This overlap may contribute to the preferential detection of class C‐like sequences in previous Antarctic metagenomic surveys [19].

### Structural diversity and conserved catalytic features of Antarctic beta‐lactamases

The 1,916 putative beta‐lactamases (BLAs), defined according to combined sequence and structural criteria, were classified into 986 SBLs and 930 MBLs. Among SBLs, 512 sequences corresponded to Class A, 77 to Class C, and 397 to Class D, with amino acid identities ranging from 42.1% to 100% relative to reference BLDB proteins. Six Class A sequences shared 97.9%–100% identity with known BLDB entries (five TEM variants and CARB‐16), and one Class D sequence exhibited 99.6% identity to OXA‐455. These high‐identity sequences likely represent widely distributed BLA variants that have been reported in both clinical and environmental contexts, including plasmid‐associated forms such as TEM‐116 [24, 25].

Within MBLs, 263 sequences were assigned to subclass B1, two to subclass B2, and 665 to subclass B3, with sequence identities ranging from 41.3% to 89.5% relative to BLDB entries (Table S3). In parallel, 127 non‐beta‐lactamase orthologs from the PBP‐like superfamily were identified (33 EstU1‐like, 86 PBP‐5‐like, and 8 PBP‐A‐like), together with 565 orthologs from the MBL‐fold superfamily, including type II glyoxylases, methyl‐parathion hydrolases, PQQ‐biosynthesis protein B, pyridoxolactonases, and ribonuclease Z‐like proteins (Table S4). These distributions further support the capacity of the workflow to discriminate BLAs from closely related proteins within the same structural superfamilies.

To assess whether predicted BLAs retained canonical catalytic features, we examined conserved motifs described for each class across sequences within the 40%–60% identity range. Forty‐six out of 51 sequences of class A (40%–50% identity) possessed the S(70)XXK, S(130)XN, and K(234)TG active site motifs, together with E166 (Figure S3). The differences were observed in the catalytic motif K(234)TG, where three sequences displayed the motif RIG, while two sequences exhibited the motif NTG. Also, the six class C sequences (50%–60% identity) comprised the S(64)XXK, Y(150)XN, and K(315)T/SG active site motifs (Figure S4). Furthermore, 49 out of 55 sequences of class D (44%–50% amino acid identity) exhibited the S(70)TFK, S(118)XV, Y/F(144)GN, and K(216)T/SG active site motifs (Figure S5). Differences were mainly identified at valine 120 within the S(118)XV motif, with five sequences showing leucine at position 120 and one sequence showing isoleucine.

For MBLs, 22 of 23 subclass B1 sequences (47%–50% identity) comprised the H(116)‐H(118)‐H(196) and D(120)‐C(221)‐H(263) zinc‐binding residues (Figure S6), with one sequence having a Tyr instead of a His residue at position 196. Additionally, 26 out of 30 sequences of subclass B3 (40%–50% amino acid identity range) contained the H(116)‐H(118)‐H(196) and D(120)‐H(121)‐H(263) zinc‐binding residues (Figure S7), where four sequences exhibit a non‐conserved substitution at serine 221 (Arg, Met, Gly, or Cys). Finally, in subclass B2, only two sequences were identified in this study, both of which contained the D(120)‐C(221)‐H(263) zinc‐binding residues. Therefore, most identified BLAs, including those with low sequence identity to reference proteins, exhibit the key motifs typical for the respective structural class. At the highest sequence identity thresholds defined for each class, at least 91% conservation was observed across all catalytic residues.

To further evaluate functional coherence, we performed multidimensional function profiling using the AnnoPRO framework [26]. Each protein was represented as a 6109‐dimensional probability vector corresponding to Gene Ontology terms (molecular function, cellular compartment, and biological process), and pairwise similarities were calculated using cosine distance, followed by UMAP dimensionality reduction (Figure S8). SBLs formed well‐defined clusters distinct from both PBP‐like and MBL‐fold‐like non‐beta‐lactamase proteins, with clear separation among Classes A, C, and D. Carboxylesterases, PBP‐5‐like, and PBP‐A‐like also formed distinct functional clusters, indicating that functional embedding distinguishes SBLs from related PBP‐like proteins independently of the sequence and structural features used for initial identification.

In contrast, most members of the broader MBL‐fold‐like superfamily displayed overlapping functional profiles, except for ribonuclease Z‐like proteins, which formed a distinct cluster. This observation underscores the greater functional diversity within the MBL‐fold superfamily and highlights the importance of integrating sequence, structural, and functional information to resolve orthologous groups within this protein family accurately.

Together, these analyses demonstrate that Antarctic beta‐lactamases encompass substantial class diversity while maintaining conserved catalytic architectures and coherent functional profiles, even at relatively low levels of sequence identity.

### Phylogenetic relationships and structural clustering of Antarctic beta‐lactamases

We next examined the phylogenetic relationships and structural diversity of Antarctic BLAs relative to reference beta‐lactamases and to non‐beta‐lactamase proteins from the PBP‐like and MBL‐fold‐like superfamilies (TNs). Antarctic BLAs and their closest reference sequences within each structural cluster were clustered at 90% sequence identity and bidirectional coverage to define representative sets. An analogous approach was applied to Antarctic proteins clustering with true negatives.

This strategy yielded representative Antarctic BLAs distributed across classes A (128), B (233; including 53 B1, 1 B2, and 179 B3), C (11), and D (72), together with 23 Antarctic PBP‐like proteins and 116 MBL‐fold‐like non‐beta‐lactamase proteins. These were analyzed alongside 587 BLDB reference sequences and 14 true‐negative representatives. Phylogenetic trees were constructed starting from multiple sequence alignments (MSAs), incorporating structural cluster information derived from the Foldseek‐based workflow (Figures 3 and 4).

**Figure 3:**
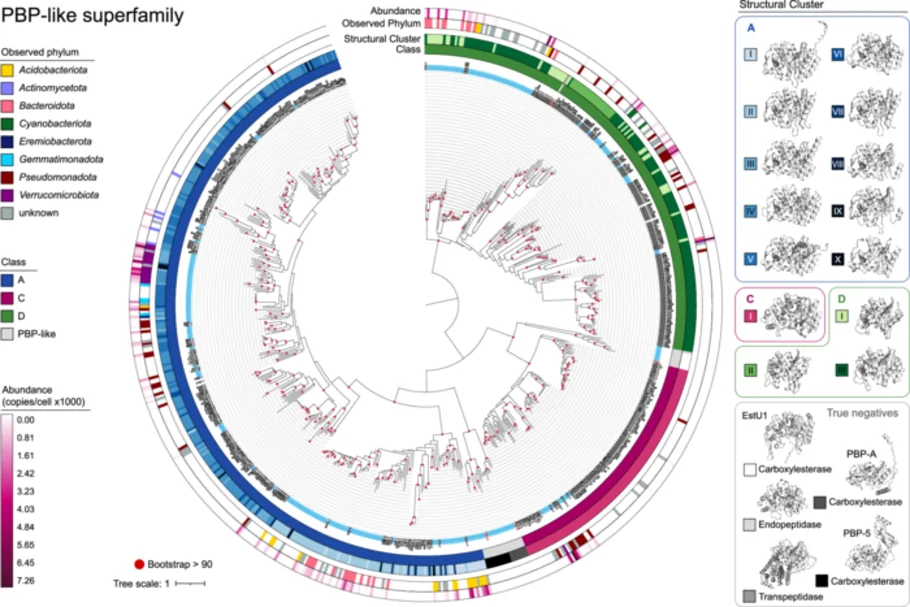
Phylogenetic relationships, structural clusters, and abundance patterns of Antarctic PBP‐like beta‐lactamases. Maximum‐likelihood tree showing representative Antarctic class A, C, and D beta‐lactamases and related PBP‐like superfamily proteins. Red, black, and blue tip labels indicate reference non‐beta‐lactamase proteins, reference beta‐lactamases, and Antarctic beta‐lactamase orthologs, respectively. Structural clusters were defined using LDDT ≥ 0.75 and TM‐score ≥ 0.75 thresholds, and representative structures are shown for each cluster. Outer rings indicate beta‐lactamase class, structural cluster, observed phylum, and cumulative abundance across all sites. Beta‐lactamase gene abundance and taxonomic affiliation were inferred from metagenomic reads mapped to each beta‐lactamase. Red circles indicate bootstrap support >90.

**Figure 4:**
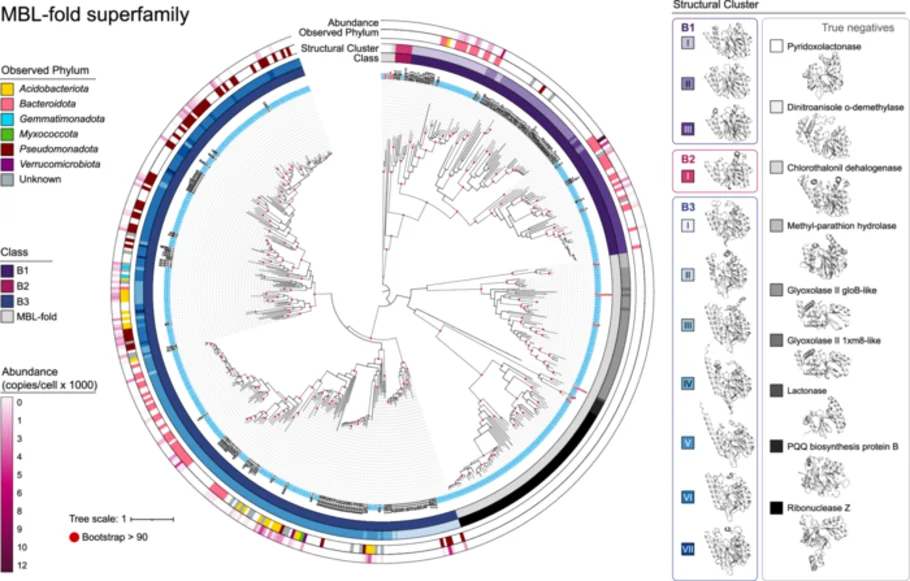
Phylogenetic relationships, structural clusters, and abundance patterns of Antarctic MBL‐fold beta‐lactamases. Maximum‐likelihood tree showing representative Antarctic class B beta‐lactamases and related MBL‐fold superfamily proteins. Red, black, and blue tip labels indicate reference non‐beta‐lactamase proteins, reference beta‐lactamases, and Antarctic beta‐lactamase orthologs, respectively. Structural clusters were defined using LDDT ≥ 0.75 and TM‐score ≥ 0.75 thresholds, and representative structures are shown for each cluster. Outer rings indicate beta‐lactamase subclass, structural cluster, observed phylum, and cumulative abundance across all sites. Beta‐lactamase gene abundance and taxonomic affiliation were inferred from metagenomic reads mapped to each beta‐lactamase. Red circles indicate bootstrap support >90.

Regarding the PBP‐like superfamily, as expected, reference beta‐lactamase sequences from classes A, C, and D formed deeply separated monophyletic clades, including Antarctic orthologs (Figure 3). The highest sequence diversity was observed in class A, followed by class D and then class C. Accordingly, a markedly higher number of structural clusters was found for class A (10), compared with class D (3) and class C (1). Conversely, all the non‐beta‐lactamase PBP‐like proteins, except for BlaR1 (class D), formed separate clades from beta‐lactamases. Regarding BlaR1, this result is consistent with previous phylogenetic analyses within the PBP‐like superfamily, indicating that this protein may represent a paralog that retains structural similarity to class D beta‐lactamases but has lost hydrolytic activity, evolving to act as a beta‐lactam sensor and signal transducer [12].

Inside class A, two major clades were observed. A more numerous one, including most of the references plus some Antarctic sequences, and a smaller one, including a higher proportion of Antarctic sequences. This separation is supported by structural clustering: the minor clade primarily included sequences from structural clusters I, II, and III, whereas the major clade was composed mainly of clusters IV, V, and VI. In class C, only one structural cluster was observed, indicating greater homogeneity within this group. In class D, three structural clusters were observed, with I and III intermingled in the tree and II more separated, all of which included Antarctic orthologs.

For all the classes, some Antarctic sequences were grouped into clades distinct from BLDB references, which could represent novel beta‐lactamase families (Figure 3). For class A beta‐lactamases, most monophyletic clades consist exclusively of Antarctic sequences, sharing 41.1% to 59.8% sequence identity with their closest references. In contrast, clades that share a common ancestor with known references mostly exhibit <70% identity, except for one clade of four sequences related to RATA‐1, which shows 80.92%–86.51% identity. RATA‐1 is a class A beta‐lactamase identified from *s_Riemerella anatipestifer* isolates, with most of its known variants reported in marine *o_Flavobacteriaceae* (*p_Bacteroidota*) [27]. This finding indicates its presence in soil‐associated environmental bacteria of the same phylum.

For Class C BLAs, the monophyletic clades primarily group with known references, including LRA18‐1, IDC‐1, MYCC1‐1, LRA10‐1, and UCB‐1. Notably, two Antarctic sequences share at least 83% identity with IDC‐1, an integron‐derived cephalosporinase previously detected by functional metagenomics in river sediments contaminated with municipal and hospital sewage [28]. Within class D, the largest monophyletic clade, composed solely of Antarctic sequences, comprises 30 BLAs with 48.3%–55.1% identity to the closest references, potentially constituting a novel family. Another clade of four Antarctic sequences clustered with OXA‐1091 (70%–74% sequence identity), a beta‐lactamase detected in the non‐clinical bacterium *s_Bradyrhizobium* sp. BTAi, which exhibits activity against oxacillin and imipenem [29].

Concerning the MBL‐fold‐like superfamily, all Antarctic sequences and BLDB reference sequences from subclasses B1, B2, and B3 clustered into separate clades compared to the non‐beta‐lactamase proteins within the superfamily (Figure 4). Subclass B1 beta‐lactamases included a large monophyletic clade of 19 Antarctic sequences sharing 48.5%–58% identity with their closest references, corresponding to a putative novel family. A distinct clade of eight sequences is closely related to MYO‐1 (70%–88% identity), a beta‐lactamase identified in *s_Myroides odoratimimus* able to hydrolyze imipenem [30]. Additionally, we identified novel Antarctic variants of CHM‐1 and CEMC19‐1 BLAs, and a highly divergent Antarctic BLA sharing a common ancestor with the CAM‐1 carbapenemase found in *s_Pseudomonas aeruginosa* [31].

For subclass B3, the largest monophyletic clade contains 28 Antarctic BLAs showing 63.4%–69.4% identity with their nearest reference (LRA12‐1) and belonging to structural cluster IV. Remarkably, LRA12‐1 was identified through functional metagenomics in a remote Alaskan soil sample [32]. In addition, we found a clade of 18 Antarctic BLAs related to CRD3‐1 (77%–84.19% identity), a BLA previously found in *g_Erythrobacter* species that displays high levels of imipenem‐hydrolyzing activity [33]. Additionally, we observed a putative novel family forming a monophyletic clade comprising six sequences that share a common ancestor with SIE‐1, first identified in *s_Sphingobium indicum*, which hydrolyzes carbapenems and cephalosporins and exhibits the peculiarity of having a Glu residue in the active site (EHH/DHH active‐site motif) [34]. We also observed an additional clade comprising mostly Antarctic BLAs related to FEZ‐1 and B3SU2‐1, initially identified in environmental *g_Flavobacterium* species [35].

The single Antarctic subclass B2 beta‐lactamase was phylogenetically distant from its closest reference (CphA‐11), consistent with a divergent lineage.

Overall, phylogenetic and structural analyses reveal extensive diversification of beta‐lactamases across Antarctic soils, including multiple monophyletic clades composed exclusively of Antarctic sequences and several lineages sharing moderate similarity with previously described environmental enzymes. These results highlight the broad evolutionary breadth of Antarctic BLAs while reinforcing the structural coherence that underlies their classification.

### Biogeographic patterns and taxonomic affiliations of Antarctic beta‐lactamases

We analyzed the abundance of BLA genes across the 81 soil metagenomes to determine their geographic distribution. Overall, MBLs accounted for 53% of the total abundance across all the sites, whereas serine beta‐lactamases (SBLs) comprised 47%. Subclass B3 was the most abundant group (39.42%), followed by class A (28.09%), class D (14.40%), and subclass B1 (13.41%) (Figure 5A).

**Figure 5:**
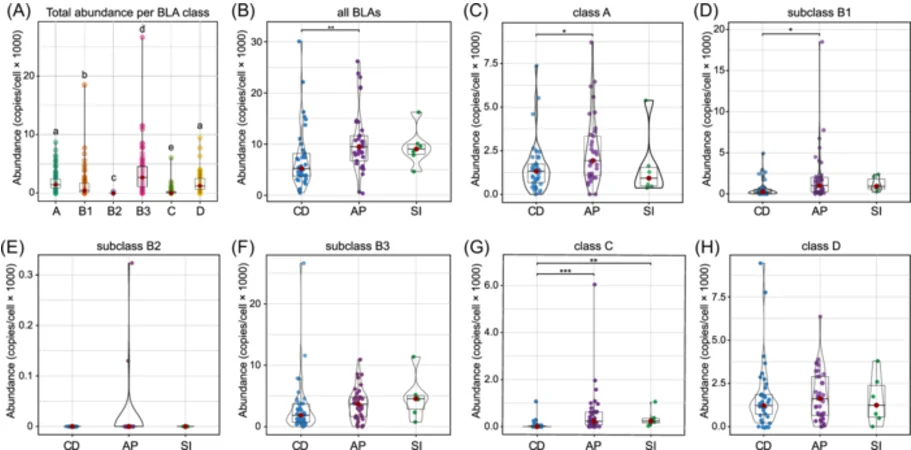
Relative abundance of Antarctic beta‐lactamases across soil ecotypes. Distribution of beta‐lactamase abundance values, expressed as copies per cell × 1000, across cold desert (CD), Antarctic Peninsula and surrounding islands (AP), and Subantarctic Islands (SI) soils. (A) Total abundance by beta‐lactamase class and subclass across all Antarctic and Subantarctic sites. Statistical differences were assessed using the non‐parametric Friedman test, followed by Durbin–Conover pairwise comparisons; different letters indicate significant differences among groups (pFDR‐adjusted < 0.001). (B) Total beta‐lactamase abundance by soil ecotype. (C–H) Beta‐lactamase abundance by class or subclass across soil ecotypes. For panels (B)–(H), statistical differences were assessed using the Kruskal–Wallis test followed by Dunn′s pairwise test. Significance is indicated as *pFDR‐adjusted <0.05, **pFDR‐adjusted <0.01, and ***pFDR‐adjusted <0.001.

Soils from AP exhibited a higher total BLA abundance than CD soils (Figure 5B). At the class and subclass levels, A, C, and B1 followed this same pattern, whereas no significant differences were observed among regions for class D, subclass B2, or subclass B3 (Figure 5C–H).

To investigate the taxonomic affiliations of Antarctic BLAs, we applied two complementary approaches. First, metagenomic reads mapping to BLA genes were extracted and taxonomically classified using RAT. Then, the BLA gene received the taxonomic classification of the mapping reads using an LCA criterion (Figure 6A).

**Figure 6:**
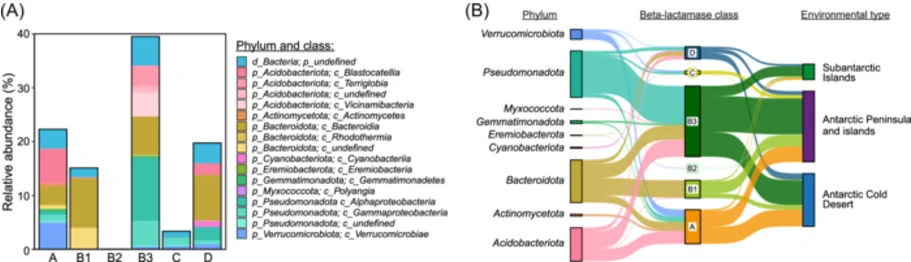
Taxonomic distribution of Antarctic beta‐lactamases. (A) Taxonomic assignment of beta‐lactamase‐coding genes based on metagenomic reads mapped to beta‐lactamase sequences and resolved using a last common ancestor approach. Bars show total relative abundance across all sites, grouped by beta‐lactamase class or subclass and predicted taxonomic affiliation. (B) Sankey plot showing the association between predicted host phyla, beta‐lactamase classes or subclasses, and environmental types for beta‐lactamases detected in assembled contigs.

Among SBLs, class A beta‐lactamases were primarily associated with *p_Acidobacteriota* (*c_Blastocatellia*), *p_Verrucomicrobiota* (*c_Verrucomicrobiae*), and *p_Bacteroidota* (*c_Bacteroidia*). Class C sequences were mainly linked with *p_Pseudomonadota* (predominantly *c_Gammaproteobacteria*), and *p_Verrucomicrobiota* (*c_Verrucomicrobiae*), whereas class D BLAs were related to *p_Bacteroidota* (*c_Bacteroidia*), *p_Pseudomonadota* (*c_Alphaproteobacteria*), and *a_Acidobacteriota* (*c_Blastocatellia*) (Figure 6A).

Within MBLs, subclass B1 was predominantly assigned to *p_Bacteroidota* (*c_Bacteroidia*), subclass B2 to sequences of undetermined phyla, and subclass B3 to *p_Pseudomonadota* (*c_Alphaproteobacteria* and *c_Gammaproteobacteria*), *p_Acidobacteriota* (*c_Terriglobia* and *c_Vicinamibacteria*), and *p_Bacteroidota* (*c_Bacteroidia*) (Figure 6A). Together, these results provide a broad overview of the potential taxonomic distribution of beta‐lactamases across Antarctic soils.

In a second approach, we examined the predicted taxonomic origins of reconstructed Antarctic BLAs within the phylogenetic trees (Figures 3 and 4), integrating relative abundance information.

For class A, the highest abundance corresponded to sequences from *p_Acidobacteriota* (*f_Pyrinomonadaceae*), forming clades related to ASU1‐1, a beta‐lactamase previously identified in environmental functional metagenomic libraries [36]. Additional Antarctic BLAs formed clades related to CME‐3 and CME‐4 within *p_Bacteroidota* (*f_Weeksellaceae*), and to RATA‐1 and VEB‐9 among *f_Flavobacteriaceae* and related lineages. Notably, a substantial fraction of class A BLAs belonged to monophyletic clades composed exclusively of Antarctic sequences, particularly within *p_Verrucomicrobiota* (*o_Chthoniobacterales*) and diverse *p_Pseudomonadota* genera, consistent with lineage‐specific diversification.

For class C, the most abundant Antarctic BLAs were affiliated with *p_Pseudomonadota* (g_*Pseudomonas_E* and *f_Rhodanobacteraceae*) and *p_Verrucomicrobiota* (*f_UBA10450*, *o_Chthoniobacterales*), sharing ancestry with UCB‐1, identified in a functional metagenomic library of agricultural and plant‐associated soil [37], and LYL‐1 from *s_Lysobacter lactamgenus*, a bacterium known for cephalosporin biosynthesis [38].

Within class D, abundant OXA‐like proteins were associated mainly with *p_Pseudomonadota* (including *g_Bradyrhizobium* and members of *f_Burkholderiaceae* and *f_Rhodanobacteraceae*), as well as with NOD‐1‐like enzymes from *p_Bacteroidota* and *p_Cyanobacteriota* lineages. Other abundant class D beta‐lactamases were related to CPD‐1 from *s_Chitinophaga* sp. (*p_Bacteroidota*, *f_Chitinophagaceae*) and OXA‐347 and OXA‐209 found in *s_Bacteroides* spp. [29].

Among MBLs, subclass B3 displayed substantial taxonomic breadth. A prominent clade corresponded to highly abundant sequences from *p_Acidobacteriota*, particularly within *o_Vicinamibacterales*, *o_Bryobacterales*, and *o_Acidoferrales*, consistent with previously noted lineage‐specific diversification.

We also observed a high abundance of B1 enzymes, mainly from *p_Bacteroidota* (g_*Ferruginibacter* and *f_Cyclobacteriaceae*), sharing ancestry with CAM‐1, a carbapenemase found in *s_P. aeruginosa* but proposed to have originated in *p_Bacteroidota* [31, 39]. Also, we identified another BLA from Bacteroidota (*f_Spirosomataceae*; *g_Spirosoma*) related to FIA‐1, identified in the genome from *s_Fibrella aestuarina* BUZ 2 and capable of hydrolyzing imipenem [30]. Additionally, we observed a high abundance of Antarctic BLAs (most from *f_Flavobacteriaceae*) sharing ancestry with CHM‐1, CEMC19‐1, and MYO‐1.

The B2 beta‐lactamase, distantly related to CphA, was found in very low abundance. In contrast, B3 beta‐lactamases were more diverse and abundant, with the representation among *p_Pseudomonadota* (g_*Rhizomicrobium*), *p_Verrucomicrobiota* (*o_Limisphaerales*), and *p_Acidobacteriota* (*o_Terriglobales*, *o_Bryobacterales*, and *o_Acidoferrales*), showing evolutionary relationships with FEZ‐1, B3SU2‐1, and AM1‐1. Enzymes from *p_Bacteroidota* (mostly from *f_Chitinophagaceae*) were also identified in high abundance, displaying similarity to LRA12‐1. Furthermore, we observed in high abundance enzymes from *p_Acidobacteriota* (*c_Vicinamibacterales*, *f*_*Fen‐336*, *f_UBA2999*, and *f_SCN‐69‐37*) and *p_Pseudomonadota* (*f_Woeseiaceae*, *f_Xanthomonadaceae*, and *f_Sphingomonadaceae*) clustered closely to SIE‐1 and THIN‐B, while others from *p_Gemmatimonadota* (*c_Gemmatimonadetes*) were related to ALG11. Finally, several *p_Pseudomonadota* BLA sequences affiliated with *g_Sphingomicrobium*, distantly related to CRD3‐1, were detected in high abundance.

Overall, these analyses reveal that Antarctic beta‐lactamases exhibit marked regional variation in abundance and broad taxonomic distribution, encompassing diverse bacterial phyla and including several lineage‐specific clades that appear enriched in Antarctic soils. To minimize confounding by unequal sample numbers across ecotypes, we also calculated per‐region normalized abundance averages for each beta‐lactamase, which revealed ecotype‐specific abundance patterns within both the PBP‐like and MBL‐fold superfamilies (Figures S9 and S10).

### Genomic context and host distribution of Antarctic beta‐lactamases

To investigate the genomic context of Antarctic BLAs, we analyzed assembled contigs and metagenome‐assembled genomes (MAGs) recovered from the 81 metagenomes. In total, 36.8 million contigs were assembled (median N50 = 1143 bp), and binning resulted in 3576 MAGs. MAG taxonomic assignments were largely consistent with taxa identified from unassembled reads and included several poorly described lineages (Table S5).

A total of 470 BLAs were identified in contigs and 175 in MAGs (Figure S11). Consistent with read‐based abundance estimates, subclass B3 and class A beta‐lactamases were the most frequently detected in both contigs and MAGs.

At the contig level (Figure 6B), class A BLAs were associated with the broadest range of phyla, primarily *p_Acidobacteriota*, *p_Bacteroidota*, and *p_Verrucomicrobiota*. Class C sequences were mainly affiliated with *p_Pseudomonadota* and *p_Verrucomicrobiota*, whereas class D BLAs were associated with *p_Bacteroidota*, *p_Acidobacteriota*, and *p_Pseudomonadota*. Some phylum‐specific associations were observed: class A BLAs were uniquely detected in *p_Actinomycetota*, and class D BLAs were uniquely identified in *p_Cyanobacteriota*. Among MBLs, subclass B3 exhibited the broadest phylogenetic distribution, predominantly within *p_Pseudomonadota*, *p_Acidobacteriota*, and *p_Bacteroidota*. Subclass B1 was largely restricted to *p_Bacteroidota*, with occasional representation in *p_Acidobacteriota*, whereas subclass B2 was detected only in *p_Verrucomicrobiota*. Notably, Verrucomicrobiota harbored five of the six BLA classes and subclasses, representing the highest class diversity within a single phylum.

Geographically, contigs encoding class A, class D, and subclasses B1 and B3 BLAs were detected across all three regions. In contrast, class C BLAs were restricted to Antarctic Peninsula and continental desert soils, while subclass B2 was detected only in AP soils, which uniquely contained representatives of all six classes and subclasses.

Within MAGs (Figure 7; Table S5), class A enzymes were distributed across the broadest range of phyla, predominantly *p_Verrucomicrobiota*, *p_Acidobacteriota*, and *p_Bacteroidota*. Class C was restricted to *p_Pseudomonadota* and *p_Verrucomicrobiota*, whereas class D spanned five phyla, with *p_Acidobacteriota*, *p_Pseudomonadota*, and *p_Bacteroidota* most prevalent. Among MBLs, subclass B3 again showed the broadest host range, mainly within *p_Pseudomonadota*, *p_Acidobacteriota*, and *p_Bacteroidota*. Subclass B1 was confined to *p_Bacteroidota*, and subclass B2 was detected only in MAGs not meeting the ≥50% completeness and <10% contamination criteria, and was, therefore, excluded from further analysis. Notably, two *p*_*Planctomycetota* MAGs encoded class A BLAs, and one *p*_*Myxococcota* MAG encoded a subclass B3 BLA, although these phyla were not associated with BLAs at the contig level.

**Figure 7:**
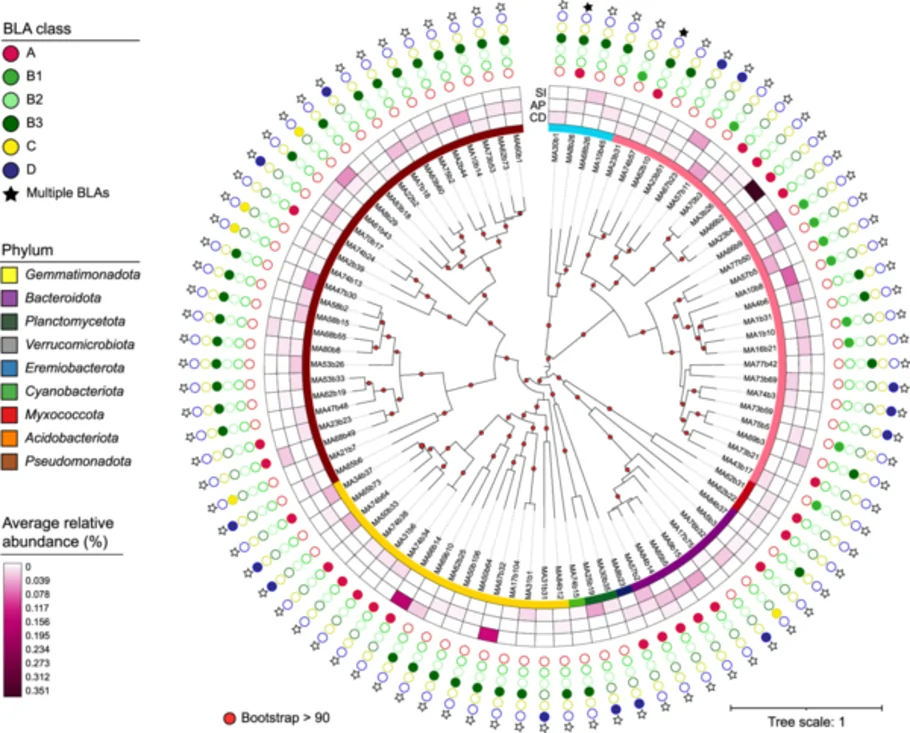
Phylogenetic distribution and regional abundance of beta‐lactamase‐containing metagenome‐assembled genomes. Midpoint‐rooted maximum‐likelihood phylogenetic tree inferred from the multiple sequence alignment of Genome Taxonomy Database (GTDB) bacterial marker genes for MAGs containing beta‐lactamase genes. The inner colored ring indicates the MAG phylum. Heatmap rings show the mean relative abundance of each MAG across Subantarctic Islands (SI), Antarctic Peninsula and surrounding islands (AP), and cold desert (CD) soils. Outer symbols indicate the presence of beta‐lactamase genes from each class or subclass. Black stars indicate MAGs encoding multiple beta‐lactamases. Red circles indicate bootstrap support >90.

Consistent with contig‐based analyses, MAGs affiliated with *p_Pseudomonadota*, *p_Bacteroidota*, and *p_Verrucomicrobiota* harbored the highest diversity of BLA classes (four out of six classes each). In two cases, individual MAGs encoded multiple BLAs from different structural classes: MA8b26 (*p_Gemmatimonadota*) and MA74b57 (*p_Bacteroidota*), each of which contained both a class A and a subclass B3 enzyme. More broadly, MAGs belonging to the same phylogenetic lineages tended to encode BLAs from the same structural classes, suggesting lineage‐associated distribution patterns.

Overall, these results demonstrate that Antarctic beta‐lactamases are broadly distributed across assembled genomes and contigs, spanning multiple bacterial phyla and exhibiting consistent host associations across structural classes. Beyond host distribution, understanding the genomic context of these beta‐lactamase genes is essential to evaluate their potential mobility. We therefore examined the association of Antarctic BLAs with mobile genetic elements (MGEs) to assess their possible capacity for horizontal transfer.

### Antarctic beta‐lactamases found associated with MGEs are limited, although likely underestimated due to assembly constraints

Detecting MGEs in metagenomic assemblies remains challenging, particularly when relying on short‐read data, due to limited contiguity and fragmentation of assembled sequences. These constraints reduce the ability to resolve full genetic contexts and may limit detection sensitivity for MGEs in environmental metagenomes [40]. Despite these limitations, we used state‐of‐the‐art bioinformatic tools to identify potential MGEs associated with BLA genes, including integrons, prophages, and transposable elements. Putative plasmid sequences were not considered due to the high false‐positive rates reported for plasmid prediction pipelines in metagenomic datasets [41].

We identified one class D OXA‐205‐like beta‐lactamase located within a complete integron in a *p_Pseudomonadota* contig (*f_Burkholderiaceae*), as well as four beta‐lactamase genes embedded in CALIN‐type integrons (Figure S12). Among these, one corresponded to a class D OXA‐1172‐like enzyme, whereas the others represented putative novel variants, including a class A enzyme (62.5% identity to GES‐41), a class C enzyme (83.5% identity to IDC‐1), and a class D enzyme (52% identity to OXA‐1089), associated with *p_Pseudomonadota* and *p_Acidobacteriota* lineages.

Analysis of insertion sequence proximity revealed additional associations. A class D BLA gene (47.4% identity against NOD‐1) was observed in the vicinity of an IS982 in a *p_Acidobacteriota* contig from *o_Pyrinomonadales*. Additionally, a class C BLA gene (61.2% identity to LRA10‐1) was identified near an IS21 in a *p_Pseudomonadota* contig from *c_Alphaproteobacteria*, and a subclass B2 BLA gene (61.9% identity to CVI‐1) was observed near an IS200/IS605 element in a *p_Verrucomicrobiota* contig from *o_Chthoniobacterales*.

No associations between beta‐lactamase genes and predicted prophages were detected. Overall, only a limited number of BLAs showed proximity to identifiable MGEs in the assembled contigs.

Given the inherent fragmentation of short‐read assemblies, these observations likely represent a conservative estimate of MGE associations in the sampled soils. Nevertheless, the detection of integron‐ and insertion sequence‐linked beta‐lactamases indicates that at least a subset of Antarctic BLAs reside within genomic contexts compatible with horizontal gene transfer.

## 3 DISCUSSION

The emergence of novel beta‐lactamases and their rapid dissemination via plasmids and other MGEs represents a critical escalation in the global AMR crisis [42, 43]. While much attention has focused on clinical settings, increasing evidence indicates that environmental microbiomes constitute a vast reservoir of beta‐lactamase diversity. In this study, we demonstrate that Antarctic and Subantarctic soil microbiota harbor a broad repertoire of beta‐lactamases spanning multiple structural classes, with a predominance of class A serine beta‐lactamases and subclass B3 metallo‐beta‐lactamases.

Structural class distribution analysis revealed that subclass B3 and class A beta‐lactamases exhibited both the highest relative abundance (39.43% and 22.3%, respectively) and the largest number of unique sequences (*n* = 665 and *n* = 512). This pattern contrasts with previous analyses of Antarctic microbial mats, where class C beta‐lactamases predominated [19], and with studies of cryosol soils reporting higher proportions of class C enzymes [44]. Such discrepancies likely reflect differences in habitat type and microbial community composition, as microbial mats and cryosols exhibit distinct taxonomic structures compared with soil environments [20, 21].

Broader environmental surveys have frequently reported a predominance of class B beta‐lactamases outside human‐associated microbiomes [45, 46], consistent with the high abundance of subclass B3 enzymes observed here. Together, these comparisons suggest that Antarctic soils align with global environmental trends in beta‐lactamase class distribution, while also harboring distinct lineage‐specific diversification.

Nonetheless, the abundance of class B beta‐lactamases in human‐derived samples may be on the rise, as suggested by recent studies [47], in response to the increased clinical use of beta‐lactam antibiotics that are not efficiently hydrolyzed by class A enzymes. This is particularly relevant, since it has been reported that class B beta‐lactamases that originate from environmental bacteria, especially those of the subclass B3, are capable of hydrolyzing all beta‐lactam antibiotics [48]. These findings highlight Antarctic environmental bacteria as a potential rich reservoir of previously uncharacterized beta‐lactamase variants, whose hydrolytic activity against clinically relevant beta‐lactam antibiotics needs to be studied.

Beyond the genomic and structural characterization, the observed geographical distribution of beta‐lactamase classes across Subantarctic islands (SI), Antarctic Peninsula and surrounding islands (AP), and cold desert (CD) soils likely reflects ecological filtering along strong environmental gradients [49]. Subantarctic and maritime Antarctic soils, which generally exhibit higher organic matter and microbial productivity, may foster more intense microbial interactions and secondary metabolite production, thereby maintaining a richer repertoire of natural resistance determinants [5]. In contrast, the nutrient‐poor and extremely oligotrophic cold deserts of continental Antarctica host simpler communities shaped by extreme abiotic stress, where beta‐lactamases could persist as multifunctional enzymes under relaxed selection [50, 51].

In terms of taxonomic distribution, consistent with previous studies, beta‐lactamases were predominantly associated with the phyla *p_Pseudomonadota* and *p_Bacteroidota*, regardless of the enzyme class or soil origin [30, 46]. Notably, we also unveiled an unprecedented diversity of BLAs from members of *p_Acidobacteriota*, being the first large‐scale BLA screening to report such an association, particularly relevant for class A beta‐lactamases. Although *p*_*Acidobacteriota* primarily comprises uncultivable representatives, recent studies have confirmed members of this phylum as ubiquitous and abundant in diverse environments [52], which highlights its potential as a gene dispersal vector. A subclass B2 beta‐lactamase (CAM‐2) has previously been reported within the chromosome of a MAG affiliated with *f_Pyrinomonadaceae*, recovered from sediments in Shenzhen Bay, China [53]. This enzyme was proposed as a potential ancestor of the clinically disseminated CAM‐1 variant found in *s_P. aeruginosa*. In the present study, 57 sequences showing 52%–83% sequence identity to CAM‐1 were identified, suggesting that Antarctic environmental *p*_*Acidobacteriota* may also serve as reservoirs for novel beta‐lactamase variants of clinical interest.

Although all the beta‐lactamases identified in this study are chromosomal, five were associated with complete or partial integrons, and three were near insertion sequences. Notably, we identified a gene encoding an OXA‐205‐like BLA in an integron, in agreement with previous reports of this enzyme associated with class 1 integrons in a clinical *s_P. aeruginosa* isolate [54], as well as in Antarctic microbial mats [55], microplastic‐associated samples along watersheds, and activated sludge from wastewater treatment plants in China [56, 57]. Additionally, two BLA genes previously associated with clinical or human‐impacted environments were identified here for the first time in a pristine, non‐human‐associated setting: the class D variant OXA‐1171/OXA‐544, initially described in clinical *s_P. aeruginosa* isolates, and a class C beta‐lactamase sharing 83.1% identity with IDC‐1, an integron‐derived cephalosporinase previously detected only in wastewater samples from Asia [28, 58].

The remaining beta‐lactamases exhibited less than 65% sequence identity to reference sequences in the Beta‐Lactamase DataBase (BDLB), indicating that they may represent previously uncharacterized families. This highlights the need for further functional characterization and assessment of their potential for horizontal gene transfer, particularly given that many beta‐lactamases acquired by clinical pathogens have originated from environmental *p_Pseudomonadota* [30, 59, 60], the same phylum to which the potentially mobile beta‐lactamases identified in this study belong.

Studies of the Antarctic resistome consistently indicate that resistance genes in remote, low‐human‐impact environments are shaped primarily by long‐standing ecological interactions rather than recent anthropogenic selection [5, 17, 61]. It has been proposed that Antarctic resistance determinants evolved in response to microbial competition and natural antibiotic production within these ecosystems. In particular, beta‐lactamases may function as defense mechanisms against beta‐lactam‐producing fungi or bacteria present in soil environments [62, 63], supporting the view that environmental beta‐lactamases reflect a long coevolutionary history between antibiotic‐producing and antibiotic‐degrading microorganisms, potentially spanning billions of years [64]. Because metadata regarding the degree of human impact were inconsistently reported across the analyzed datasets, we could not systematically evaluate the effect of local anthropogenic activity on beta‐lactamase profiles, and therefore our interpretations primarily reflect broad ecological patterns rather than site‐specific disturbance gradients.

The predominance of class A and subclass B3 enzymes observed here is consistent with their deep evolutionary origins among serine beta‐lactamases and metallo‐beta‐lactamases, respectively. Their long‐term persistence and structural conservation may contribute to their widespread distribution in low‐human‐impact environments such as Antarctica, where selective pressures are primarily ecological rather than clinical. Moreover, several class A and subclass B3 variants have been shown to possess relatively broad substrate spectra [11, 48], which could confer adaptive flexibility in heterogeneous soil environments.

Alternatively, the persistence of these enzymes in cold, nutrient‐limited ecosystems may reflect ancestral multifunctionality. Promiscuous catalytic activities within the PBP‐like and MBL‐fold superfamilies can be co‐opted for diverse physiological roles under fluctuating selective pressures [65]. For example, serine beta‐lactamases share structural features with proteins involved in cell‐wall remodeling [66, 67], while metallo‐beta‐lactamase‐fold proteins have been implicated in quorum sensing and other regulatory processes [68, 69]. This perspective is particularly relevant in Antarctic soils, where diverse homoserine lactone‐based quorum‐sensing systems have been documented as adaptive strategies in extreme environments [70]. Elucidating the native physiological roles of Antarctic beta‐lactamases will therefore be essential to understanding their ecological significance.

Advances in next‐generation sequencing and structure‐informed bioinformatics have greatly expanded the detection of resistance gene homologs in environmental microbiomes [71]. For beta‐lactamases in particular, it has been estimated that tens of thousands of variants remain undiscovered [72]. The integrative workflow applied here, combining sequence, structural, and functional similarity, enables the identification of deeply divergent orthologs while minimizing misclassification within large superfamilies. In this context, notably, metagenomic‐metatranscriptomic studies of gut microbiomes have shown that a substantial proportion of detected β‐lactam ARGs are not transcriptionally active, indicating that gene presence alone does not necessarily reflect gene expression [73]. Antarctic soils provide a valuable system for exploring the evolution of beta‐lactamase architectures under predominantly natural selective regimes.

Finally, several enzymes identified in this study originate from bacteria inhabiting permanently cold environments, where winter temperatures may reach −60°C [74]. As such, they may represent models for studying structural stability and catalytic adaptation under low‐temperature conditions. Investigating their biochemical properties could yield insights into the evolutionary constraints and flexibility of beta‐lactamase scaffolds in extreme ecosystems.

## 4 CONCLUSION

Together, our findings demonstrate that Antarctic and Subantarctic soils constitute geographically structured and phylogenetically diverse reservoirs of beta‐lactamases, dominated by class A and subclass B3 enzymes and largely encoded by members of *p_Pseudomonadota* and *p_Bacteroidota*. The integrative sequence–structure–function framework employed here revealed a wide repertoire of previously uncharacterized and deeply divergent beta‐lactamases that nonetheless retain conserved catalytic architectures, underscoring the evolutionary persistence of these enzyme families in cold environments. These results support the view that remote ecosystems contribute significantly to global resistome diversity and reflect long‐standing ecological and evolutionary processes rather than recent anthropogenic selection. Moreover, the prevalence of novel variants highlights the need for future functional characterization to determine their biochemical properties, substrate spectra, and potential for mobilization. In this context, Antarctic soils provide a valuable natural system for understanding the evolution, diversification, and functional potential of beta‐lactamases within the broader One Health framework.

## 5 METHODS

### Antarctic soil metagenome database construction

This study included 69 metagenomes downloaded from the NCBI Sequence Read Archive (SRA) database as of September 26, 2023, and 12 metagenomes sequenced by our research group. As inclusion criteria, we used all the metagenomes that matched the query “Antarctic soil metagenomes,” which included their geographical localization, effectively came from soil samples, and corresponded to shotgun metagenomic sequencing. For replicate sequence files from the same soil sample, we selected the one with the highest sequencing effort. All analyzed metagenomes correspond to soil samples; differences in the level of environmental detail reported in Table S1 reflect variation in the original metadata submitted to NCBI rather than differences in soil type. Because technical and environmental covariates were not consistently available across datasets, they could not be incorporated into the inferential models; therefore, unmodeled covariates may contribute to residual variation in community structure and beta‐lactamase abundance estimates. The metagenomes' FASTQ files were downloaded using their SRA run accession and a combination of prefetch and fasterq‐dump tools from SRA‐toolkit v2.11.3.

### Metagenomic reads quality assessment and trimming

Raw Illumina reads were quality‐checked and processed using fastp v0.23.4 [75] with default parameters to filter low‐quality reads and possible adapters. We also assessed human contamination through mapping all the metagenomes to the human reference genome GRCh38.p14 (accession: GCF_000001405.40) using bwa‐mem2 v2.2.1 [76] with default parameters and samtools v1.13 [77] to discard all the mapped reads.

### Sequence and microbial diversity analysis

Sequence reads for each metagenome were processed using SingleM pipe v0.18.3 [22] with default parameters, and then summarized using the summarize subcommand to determine operational taxonomic units (OTUs) at the species level, by clustering similar sequences at a 96.66% identity threshold for 43 essential single‐copy universal bacterial and archaeal genes (ESCGs). Next, for each ESCG, α‐diversity metrics including richness, Shannon indices, Pielou evenness and others related metrics were calculated using the custom Perl script get_indices_from_unifrac_tables.pl, where the input corresponds to the summarized by OTUs output of SingleM (SingleM summarize –unifrac‐by‐otu). When the above command is executed, 59 tables are generated, one for each of the archaeal/bacterial ESCGs. These 59 tables are used as input for the custom Perl Script, recovering all α‐diversity metrics for each ESCG, which can then be used to generate Boxplots and related statistical tests presented in Figure 1. Only 43 out of 59 ESCGs were kept for further analyses, discarding those genes where the estimated richness value equaled zero or one, and genes whose α‐diversity metric estimations fell outside the 95% confidence interval for the observed α‐diversity metrics distribution.

The Shannon Index H was calculated standardly according to the following formula:

$$H=-\sum_{x\epsilon X}p\left(x\right)\;\ln p\left(x\right),$$

where *x* corresponds to each observed OTU for the ESCG *X*, *p*(*x*) corresponds to the relative abundance of OTU *x*, and the log base is e.

The Pielou Index *J* is calculated standardly according to the following formula:

$$J=\frac{H}{\ln\left(R\right)},$$

where *H* corresponds to the Shannon Index, and *R* corresponds to the Richness for ESCG *X*, which corresponds to the number of distinct OTUs × observed for ESCG *X*.

Next, for each α‐diversity metric (richness, Pielou index, and Shannon index), we implemented a multivariate non‐parametric rank‐based analysis using the mv.Csample.test function from the MNM R package [78], to compare α‐diversity metric distributions between environmental types. This approach considers the α‐diversity metrics of all 43 ESCGs simultaneously as a single multivariate vector for each metagenome. We employed outer standardization, spatial rank scores, and a permutation‐based *p*‐value estimation (1000 permutations) (score = “r”, stand = “o”, method = “p”) to rigorously account for the non‐normal distribution and potential outliers inherent in microbial diversity data. This was followed by post‐hoc pairwise comparisons with FDR *p*‐value adjustment. Then, for visualization purposes, we implemented the ggstatsplot package [79] in R to plot α‐diversity metrics. In these plots, each data point corresponds to the mean value across all 43 ESCGs α‐diversity metric estimations for each metagenome (*n* = 1 per site), ensuring the visualization is consistent with the multivariate statistical design and free from pseudoreplication.

Additionally, SingleM results were used to determine the taxonomic profiles of the observed communities in each soil sample based on the GTDB r220 taxonomy database [80]. These profiles were used to estimate beta‐diversity (Bray–Curtis dissimilarity) at the family level among all the metagenomes and assess possible associations with the environmental type using the vegan [81] and pairwiseAdonis (https://github.com/pmartinezarbizu/pairwiseAdonis) packages in a custom R script. The family level was chosen to maximize the proportion of reads assigned to any taxa (70.80%−94.82%).

Next, we complemented the NMDS 2D projection and corresponding PERMANOVA, with a permutation‐based multivariate homogeneity of group dispersions test, to estimate if the average distances of group members to the spatial median of the group, used as group centroid, in multivariate space are different between groups. This complementary analysis was performed using the betadisper function from the vegan R package [81], considering the bias correction for unequal number of samples (type = “median,” bias.adjust = TRUE), followed by a permutation test (permutations = 999) to test the null hypothesis of homogeneity of multivariate dispersions, and post‐hoc pairwise comparisons with FDR‐adjusted *p*‐values. Distances to centroids distributions for each ecotype were plotted as a boxplot, using the ggstatsplot [79] package in R, presented in Figure S1.

NonPareil v3.401 [82] was used in kmer mode to estimate metagenomic coverage and sequencing effort for each sample. MASH v2.3 [83] was used to estimate k‐mer distance between metagenomic samples, using the options “sketch,” “dist,” and default parameters. Figures were plotted using the ggplot2 and ggstatsplot [79] packages in R.

### Metagenome assembly and protein prediction

Metagenomic trimmed reads were assembled into contigs using metaSPAdes v3.15.5 [84] with default parameters. These contigs were processed using Prodigal v2.6.3 [85] in meta mode to translate the nucleotide sequences from all metagenomic samples. Additionally, protein sequences were directly assembled from trimmed reads using PLASS [23] in assemble mode with default parameters, adjusting the –min‐length to 30 or 40 depending on the read length of the samples (100 bp or <150 bp, respectively). Only complete protein sequences identified by Prodigal and PLASS were retained for subsequent analyses. Partial protein sequences assembled by PLASS were excluded using the custom Perl script plass_orf_filter.pl.

### Beta‐lactamase protein sequence database construction and identification of (sub)Antarctic beta‐lactamases

On October 5th, 2023, 7566 BLA protein sequences were downloaded from the Beta‐Lactamase database BLDB [86], preserving the protein family and enzyme class associated with each. Additionally, six PBP‐fold superfamily and 18 MBL‐fold‐like superfamily non‐beta‐lactamase protein sequences were added as true negatives (TN DB, Table S3).

BLDB protein amino acid lengths were determined, and standard statistical metrics were calculated for the obtained length distribution (Q0 = 95, Q1 = 284, Q2 = 313, Q3 = 377, Q4 = 428, arithmetic mean = 329.336, standard deviation = 52.4, mode = 377). Based on the length distribution of BLDB proteins, PLASS‐assembled and Prodigal‐predicted metagenomic proteins were filtered by amino acid length (minimum length = 100 aa, maximum length = 500 aa) using the custom Perl script filter_by_length.pl. After applying this size filter, 98,161,403 PLASS proteins (PLASS DB) and 9,024,650 Prodigal (Meta DB) proteins were kept. Proteins from each database (BLDB, PLASS DB, and Meta DB) were redundancy filtered separately with MMSeqs2 v15.6f452 [87] in Linclust mode [88] using sequence identity ≥0.99 and bidirectional coverage ≥0.99 thresholds (‐‐alignment‐mode 3 ‐‐cov‐mode 0 ‐‐cluster‐mode 2 ‐‐realign), only keeping the representative protein sequences for further steps.

All 65,532,405 redundancy‐filtered protein sequences (5458 from BLDB, 58,058,386 from PLASS DB, 7,468,537 from Meta DB, and all 24 from TN DB) were redundancy‐filtered as a single set following the same procedure described previously. Then, the resulting 62,047,905 protein set was clustered using low stringency thresholds (sequence identity ≥ 0.2 and bidirectional coverage ≥ 0.8) with MMSeqs2, as described previously. All 267 low‐stringency clusters containing BLDB or TN DB proteins were kept for further analysis, using the custom Perl script get_relevant_clusters_list_v2.pl. Eighty‐one clusters also contained 3502 Antarctic proteins (PLASS DB or Meta DB).

All 3502 relevant Antarctic proteins selected in the previous step and 5482 redundancy‐filtered reference sequences (5458 BLDB proteins and 24 TN DB proteins) were used as input for the protein structure predictor ESMFold v1.0.3 (ESM‐2 model) [89]. Next, Foldseek v8.ef4e960 [90] was used to cluster all 8984 predicted protein structure models with highly stringent global and local structural similarity thresholds (TM ‐ Score ≥ 0.75 and LDDT ≥ 0.75) while keeping the lax sequence identity thresholds of the previous MMSeqs2 clustering (sequence identity ≥ 0.2 and bidirectional coverage ≥ 0.8) (‐‐cov‐mode 0 ‐e 1 ‐‐cluster‐mode 2 ‐‐cluster‐reassign ‐s 7.5 ‐‐tmalign‐hit‐order 3 ‐‐tmalign‐fast 0 ‐‐sort‐by‐structure‐bits 1). Then, from the 70 structural clusters obtained, 34 contained both reference and Antarctic proteins (25 clusters contained BLDB reference proteins, while 9 contained TN reference proteins).

All 3025 Antarctic protein sequences in 34 relevant structural similarity clusters were considered putative beta‐lactamases (2174) or TN non‐beta‐lactamase proteins (851). Next, the enzyme class from reference proteins in each structural cluster was inherited to putative Antarctic BLAs and TNs (no cluster contained more than one enzyme class). Similarly, redundant protein sequences from previous steps (≥0.99 sequence identity and ≥0.99 bidirectional coverage clusters) were assigned to structural clusters by reverse inheritance. Finally, the 3025 selected Antarctic proteins and all 7590 reference proteins were clustered iteratively using DIAMOND BLASTP v2.1.9 [91] in ultra‐sensitive mode until each protein belonged to a unique single‐linkage cluster (99% identity and 99% bidirectional coverage thresholds). The 4892 representatives of these clusters were selected as the final Antarctic BLA set (1916 proteins), Antarctic TN set (692 proteins), reference BLA set (2260 proteins), and reference true negatives set (24 proteins).

### Multiple amino acid sequence alignment and phylogenetic analyses

For each of the 34 previously mentioned relevant structural clusters and five more structural clusters containing only reference TNs, the final protein dataset corresponding to each of those clusters was redundancy filtered with MMSeqs2 as previously described using sequence identity ≥0.9 and bidirectional coverage ≥0.9 thresholds. Then, the obtained 1184 non‐redundant protein dataset was divided into two groups: the corresponding PBP‐like (677 proteins) and MBL‐like (507 proteins). Next, the protein sequences in each group were aligned using MAFFT v7.505 [92] in globalpair mode with 1000 iterations, followed by trimming of constant and uninformative sites based on the alninfo analysis performed by IQ‐TREE v2.2.5 [93], using the custom Perl script make_informative_msa.pl. Trimmed alignments were then used as input for maximum likelihood tree inference using IQ‐TREE (‐‐seed 139808 ‐‐bnni ‐‐allnni) with 1000 ultrafast bootstraps [94] and the ModelFinderPlus algorithm [95] to select the best amino acid substitution model for each tree according to the BIC criterion, Q.pfam+R10 for the PBP‐like superfamily tree and WAG + F + R9 for the MBL‐like superfamily tree. Phylogenetic trees visualization was performed using iTOL v7.0 [96].

### Beta‐lactamases structural alignment and visualization

All the predicted structures for the 1184 non‐redundant proteins, which were used as input for phylogenetic trees inference, were structurally aligned using US‐align v20240319 [97] (‐mol prot ‐mm 4 ‐ter 2). Structure PDB files were imported into PyMOL v3.1.3 (Schrodinger, LLC), then signal peptides detected by SignalP v6.0 [98] were removed. Finally, structure visualization cartoons were made in ray_trace_mode 3.

### Contig binning and reconstruction of metagenome‐assembled genomes (MAGs)

The trimmed reads were mapped to their respective contigs in all the samples using bwa‐mem2 for metagenomic contig binning. The SAM files were then filtered and sorted with samtools and processed with the jgi_summarize_bam_contig_depths command to obtain per‐sample contig coverage depth. These files were required as input for binning the contigs using MetaBAT2 v2.15 [99]. Using the default parameters, 3576 bins were obtained from these metagenomic samples. All the bins were quality‐checked using CheckM2 v1.0.1 [100]. Only MAGs with ≥50% completeness and <10% contamination (MiMAG criteria) [101] were retained for phylogenetic and taxonomic analyses. Under this parameter, we obtained 1523 MAGs.

### Taxonomic assignment of MAGs and metagenomic contigs

Previous BLA representatives were used to determine which contigs and MAGs contained these enzymes using DIAMOND BLASTX with default parameters. The results were filtered using a custom R script that required a minimum 70% identity and 80% coverage match, yielding 547 contigs and 90 MAGs containing BLAs. Taxonomic assignment of contigs containing BLAs was performed using the Contig Annotator Tool CAT v6.0 [102] in sensitive mode supplemented with the GTDB r220 database [80]. The taxonomy and BLA class information for all the contigs were summarized in a Sankey diagram plotted in R using the ggsankey package (https://github.com/davidsjoberg/ggsankey). High‐ and medium‐quality MAGs were also taxonomically identified using GTDB‐tk v2.3.0 in classify_wf mode with the GTDB r220 database. The MSA output of this workflow was used as input to IQ‐TREE in autodetection mode (‐‐seed 314716, best model: LG + F + R6, bootstrap: 1000) for maximum likelihood tree inference of the MAGs comprising BLAs. Tree visualization and editing were performed with ITOL.

Additionally, we mapped metagenomic reads back to BLA‐containing MAGs to calculate their average regional relative abundance (percentage of mapped reads), only considering alignments where at least 75% of the read was mapped to the reference sequence with 95% identity or more, following the recommendations of Poff et al. [103], using CoverM v0.7.0 [104] (‐p bwa‐mem2 ‐‐min‐read‐aligned‐percent 75 ‐‐min‐read‐percent‐identity 95 ‐‐proper‐pairs‐only ‐‐exclude‐supplementary ‐m relative_abundance).

### Beta‐lactamase gene taxonomic assignment and relative abundance calculation

The final set of 4176 BLA sequences was divided into six groups according to structural class (A, B1, B2, B3, C, and D). For each group, a MSA was generated using MAFFT as described previously, and profile hidden Markov models (HMMs) were constructed using HMMER v3.2.1 [105]. All BLAs of the respective class identified in taxonomically assigned contigs were subsequently realigned into their corresponding HMM to generate refined reference MSAs.

Then, each class‐specific MSA and HMM was used as input for SingleM seqs v0.18.3 [22] to find the most conserved 20 amino acid region within each class profile. The resulting HMMs were then used to build GraftM packages using GraftM v0.15.1 [106], and these were incorporated into class‐specific SingleM packages.

The six SingleM packages (one per BLA class) were used to identify BLA‐containing sequencing reads in each metagenome using SingleM in pipe mode. This class‐specific conserved‐region strategy enabled reliable detection of BLA‐associated reads independently of contig length, thereby minimizing biases introduced by short or fragmented assemblies.

All identified BLA‐containing reads for each metagenome were taxonomically assigned using the Read Annotator Tool RAT v6.0 in sensitive mode [107], supplemented with the GTDB r220 database and previously assigned contig‐level taxonomy obtained using CAT. For each read, the specific observed BLA sequence was determined using MMSeqs2 v15.6f452 in easy‐search mode (‐s 7 ‐‐alignment‐mode 3) against the curated BLA database (4,176 proteins) and the TN database (716 proteins). Stringent parameters previously recommended for ARG detection [108] were applied (e‐value ≤ 1e‐7, identity ≥ 80%, query coverage ≥ 75%, and alignment length of at least 25 amino acids). Reads failing to meet these criteria were discarded.

For each BLA gene, all taxonomic assignments derived from mapped reads were integrated using a hierarchical Last Common Ancestor (LCA) resolution procedure implemented in the custom script get_abundance_and_taxonomy.pl. Taxonomic reconciliation proceeded sequentially from the domain to the species level. At each rank (domain, phylum, class, order, family, genus, species), assignments were compared across reads; the process stopped at the highest rank showing no conflict among observed classifications. Missing taxonomic ranks were not treated as conflicts. This multi‐step consensus approach ensured conservative and robust taxonomic assignment even when BLAs were encoded on short or fragmented contigs.

The relative abundance of each BLA (copies/cell) in all metagenomes was determined according to the following equation [109]:

$$\mathrm{Abundance}=\sum_{i=1}^{n}\left(K\times \frac{\frac{\mathrm{Ni}\;\mathrm{BLAi}\;\mathrm{read}\times \mathrm{Li}\;\mathrm{read}}{\mathrm{Li}\;\mathrm{BLAi}\;\mathrm{reference}\;\mathrm{sequence}}}{\mathrm{Ncell}\;\mathrm{number}}\right)$$

where “*Ni BLAi read*” is the number of reads coding for a specific BLA, “*Li read*” is the read length, “*Li BLAireference sequence”* is the length of the nucleotide sequence of the BLA, *Ncell number* corresponds to the estimated number of cells in the metagenome, and *K* is a normalization parameter for multi‐gene antibiotic resistance mechanisms (for BLAs, *K* = 1). The number of cells in each metagenome was estimated using the SingleM microbial_fraction subcommand [110] as the quotient between the number of prokaryotic nucleotide bases and the average prokaryotic genome size. These abundance values should therefore be interpreted as standardized metagenome‐level estimates rather than direct measurements with explicitly propagated uncertainty.

Next, we calculated per‐region normalized abundance averages (expressed as copies per cell ×1000) for each individual beta‐lactamase.

### Functional prediction and similarity clustering of beta‐lactamases and related proteins

For each of the 4982 proteins in the database, functional prediction annotation based on Gene Ontology (GO) terms was performed using AnnoPRO v0.2‐alpha+2.g547770e [26]. Then, functional similarity between proteins was determined by calculating the cosine distance between GO‐term probability vectors [111] using the custom Perl script make_annopro_matrices.pl. Additionally, Uniform Manifold Approximation and Projection (UMAP) [112] was performed using all 4982 GO‐term vectors to determine functional similarity groups between proteins and plot results (n_components = 2, random_state = 42, n_neighbors = 100, min_dist = 0.1, metric =′cosine′).

### Performance evaluation of the bioinformatic workflow for structure‐guided identification of BLA from metagenomic data

We considered using two standard processes to search for BLA orthologs: MMSeqs2, using local alignments, and HMMER to perform searches using hidden Markov Models (HMMs) of BLA profiles. First, for MMSeqs2 v15.6f452, we used all complete PLASS proteins and all complete translated ORFs observed in the contigs assembled by metaSPAdes as queries to search against all BLDB targets (easy‐search mode –alignment‐mode 3) and then retrieved all 413,632 best hits according to bitscore without any filtering.

Next, for the HMMER v3.2.1 implementation, we first split BLDB into 6 fasta files, one for each BLA class (A, B1, B2, B3, C, and D). We inferred a MSA for each of them with MAFFT v7.505 (‐‐globalpair –maxiterate 1000), then constructed HMMs using hmmbuild and hmmpress, and searched for BLA orthologs in all queries described above using hmmscan against all built HMMs. All outputs were concatenated, and for each query, only the best hit according to the full‐sequence HMMER score was retained. A total of 422,398 hits were observed using this method.

Then, assuming our final database of 1916 candidate Antarctic BLAs and 692 Antarctic True Negatives candidates (not‐BLA proteins from PBP‐like and MBL‐like superfamilies) obtained by the structural alignment‐based workflow as a high‐confidence reference set to test the boundaries of search algorithms' sensitivity, we evaluated the performance of both methods, MMSeqs2 and HMMER, using successive thresholds of transformed e‐values, according to the following formula, where *T* corresponds to the transformed score and *E* to the *e*‐value: *T* = −ln(E).

For each threshold, performance metrics including sensitivity, specificity, precision, false discovery rate, and Matthews Correlation Coefficient (MCC) were calculated, and the corresponding Receiver Operating Characteristic (ROC) curves were plotted using the custom Perl scripts get_ROC_table_hmm.pl and get_ROC_table_mmseqs.pl. For all thresholds, all 1916 candidate Antarctic BLAs were always observed by both methods. Next, optimal thresholds for each method were determined according to MCC scores.

For MMSeqs2, the optimal e‐value corresponded to 4.96e‐15 (MCC = 1, FDR = 0), allowing the detection of 76,837 new potential BLAs not included in our set; and for HMMER, the optimal e‐value corresponded to 2.99e‐22 (MCC = 1, FDR = 0), allowing the detection of 80,186 potential Antarctic BLAs.

Next, we combined both lists of potential new Antarctic BLAs into a non‐redundant set of 92,283 proteins (64,740 detected by both methods, 15,446 by HMMER alone, and 12,097 by MMSeqs2 alone). This set was locally aligned against our high‐confidence reference set, supplemented with all BLDB reference BLAs and the 24 True Negative reference proteins using MMSeqs2 (easy‐search mode –alignment‐mode 3). Best hits were selected according to bitscore and quantified.

### MGE analysis

All contigs coding for BLAs were used as input for predicting complete integrons, clusters of attC sites lacking integron‐integrases (CALIN), and integron integrases using IntegronFinder v2.0.5 [113]. Integrated prophages were predicted using the PHASTEST [114] web server (accessed November 26th, 2024) and Phigaro v2.4.0 [115]. Complete and partial insertion sequences were predicted using ISEScan v 1.7.2.3 [116]. Predicted ISs located ≤5000 bp upstream or downstream from BLA genes were considered relevant hits. MGEs' genomic features were identified and annotated using Bakta v1.10.1 [117] in meta mode using the full Bakta database v5.1 (https://zenodo.org/records/10522951).

## AUTHOR CONTRIBUTIONS

**José Coche‐Miranda**: Conceptualization; methodology; software; formal analysis; investigation; data curation; visualization; writing—original draft; writing—review and editing. **Patricio Arros**: Methodology; software; formal analysis; investigation; data curation; visualization; writing—review and editing. **Nicolás Canales**: Investigation; data curation; writing—review and editing. **Camilo Berríos‐Pastén**: Investigation; data curation; writing—review and editing. **Gastón Azziz**: Resources; data curation; writing— review and editing. **Rosalba Lagos**: Supervision; writing—review and editing. **Julieta Orlando**: Resources; supervision; writing—review and editing. **Francisco P. Chávez**: Conceptualization; supervision; funding acquisition; writing—review and editing. **Andrés E. Marcoleta**: Conceptualization; methodology; supervision; project administration, funding acquisition; writing—original draft; writing—review and editing.

## CONFLICT OF INTEREST STATEMENT

The authors declare no conflicts of interest.

## ETHICS STATEMENT

No animals or humans were involved in this study.

## Supporting information


**Figure S1:** NMDS ordination quality and beta‐dispersion analysis of Antarctic soil microbial communities.
**Figure S2:** Benchmarking of sequence‐based beta‐lactamase searches against the structurally curated candidate set.
**Figure S3:** Conservation of catalytic and other relevant motifs in divergent Antarctic class A beta‐lactamases.
**Figure S4:** Conservation of catalytic and other relevant motifs in divergent Antarctic class C beta‐lactamases.
**Figure S5:** Conservation of catalytic and other relevant motifs in divergent Antarctic class D beta‐lactamases.
**Figure S6:** Conservation of catalytic and other relevant motifs in divergent Antarctic subclass B1 metallo‐beta‐lactamases.
**Figure S7:** Conservation of catalytic and other relevant motifs in divergent Antarctic subclass B3 metallo‐beta‐lactamases.
**Figure S8:** Functional similarity clustering of Antarctic candidate beta‐lactamases and related true‐negative proteins.
**Figure S9:** Distribution of beta‐lactamase classes across assembled metagenomic contigs and metagenome‐assembled genomes.
**Figure S10:** Regional normalized abundance of PBP‐like beta‐lactamases across Antarctic soil ecotypes.
**Figure S11:** Regional normalized abundance of MBL‐fold beta‐lactamases across Antarctic soil ecotypes.
**Figure S12:** Genomic contexts of Antarctic beta‐lactamases associated with putative mobile genetic elements.

**Table S1:** Antarctic and Subantarctic soil metagenomes analyzed in this study.
**Table S2:** PBP‐like and MBL‐like proteins lacking beta‐lactamase activity used as negative controls for the search of Antarctic beta‐lactamase orthologs.
**Table S3:** Predicted beta‐lactamase orthologs in the Antarctic and Subantarctic soil metagenomes.
**Table S4:** Putative orthologs of true negatives from the PBP‐like and MBL‐fold‐like superfamilies by protein class identified in 81 Antarctic metagenomes using the workflow proposed in this study.
**Table S5:** Assembled contigs and MAGs from Antarctic and Subantarctic soil metagenomes.

## Data Availability

The data that support the findings of this study are openly available in NCBI at https://www.ncbi.nlm.nih.gov/, reference number PRJNA1425240. The metagenomic data used in this study come from two sources. 69 of them were downloaded from the SRA archive (NCBI database). The rest of the metagenomes were generated from Antarctic soil samples acquired by the research groups involved in this study, and the reads were deposited in the NCBI database under the BioProject number PRJNA1425240 (https://www.ncbi.nlm.nih.gov/bioproject/PRJNA1425240). The accession numbers and relevant metadata are included in Table S1. The Antarctic beta‐lactamase and True Negatives collection generated by this research is available at https://github.com/mudamaid62/Coche_2026_BLA_database. All custom Perl, Python, and R scripts, and raw data used for this research are available in the following Github repository: https://github.com/mudamaid62/Coche2026imetaomics. Supplementary materials (figures, tables, graphical abstract, slides, videos, Chinese translated version, and updated materials) may be found in the online DOI or iMetaOmics http://www.imeta.science/imetaomics/.
