# Supplementary material for: Antarctic soil microbiomes encode structurally conserved and phylogenetically diverse beta‐lactamases
Source: IMetaOmics. 2026 Jul 4;3(3):e70118. doi: 10.1002/imo2.70118 (PMC13674405; doi:10.1002/imo2.70118)
Supplement: Supplementary file 1 [file IMO2-3-e70118-s002.docx]

**Supporting information to**

**Antarctic Soil Microbiomes Encode Structurally Conserved and Phylogenetically Diverse Beta-Lactamases**

**Running title:** Antarctic beta-lactamases

José Coche-Miranda^1#^, Patricio Arros^1#^, Nicolás Canales^1^, Camilo Berríos-Pastén^1^, Gastón Azziz^2,3^, Rosalba Lagos^1^, Julieta Orlando^4,5^, Francisco P. Chávez^6^, Andrés E. Marcoleta^1*^

^1^Grupo de Microbiología Integrativa, Laboratorio de Biología Estructural y Molecular BEM, Departamento de Biología, Facultad de Ciencias, Universidad de Chile, Santiago, 7800003, Chile.

^2^Microbiology Laboratory, Faculty of Agronomy, Universidad de la República, Montevideo, 12900, Uruguay.

^3^Molecular Microbiology Unit, Clemente Estable Biological Research Institute, Montevideo, 11600, Uruguay.

^4^Laboratorio de Ecología Microbiana, Departamento de Ecología, Facultad de Ciencias, Universidad de Chile, Santiago, 7800003, Chile.

^5^Instituto Milenio Biodiversidad de Ecosistemas Antárticos y Subantárticos, Santiago, 7800003, Chile.

^6^Laboratorio de Microbiología de Sistemas, Departamento de Biología, Facultad de Ciencias, Universidad de Chile, Santiago, 7800003, Chile.

#These authors contributed equally: José Coche-Miranda, Patricio Arros

*Correspondence: amarcoleta@uchile.cl (Andrés E. Marcoleta)

**Figure S1. NMDS ordination quality and beta-dispersion analysis of Antarctic soil microbial communities.** (A) Stress plot of the non-metric multidimensional scaling (NMDS) ordination based on Bray-Curtis dissimilarities calculated from family-level taxonomic profiles across Antarctic and Subantarctic soil metagenomes. The stress value of 0.1156 indicates a satisfactory two-dimensional representation of the multidimensional dissimilarity matrix. (B) Distances of individual samples to the spatial median of their respective ecotype groups (Cold Desert, CD; Antarctic Peninsula, AP; Subantarctic Islands, SI), calculated using the betadisper function. Violin plots and boxplots show the distribution of distances to the group median for each ecotype. Red dots indicate group medians. Pairwise comparisons were performed using permutation-based tests with FDR correction. Significant differences between CD and AP and between CD and SI indicate heterogeneity in within-group dispersion among ecotypes.

**Figure S2. Benchmarking of sequence-based beta-lactamase searches against the structurally curated candidate set.** Receiver operating characteristic (ROC) curves showing the performance of two sequence-based strategies for beta-lactamase ortholog detection: MMSeqs2 (A) and HMMER (B). Curves show the relationship between the false positive rate and true positive rate across E-value-dependent thresholds, using the 1,916 Antarctic candidate BLAs and 692 Antarctic candidate non-BLA proteins identified by the structure-informed workflow as an internal reference set. (C) Local alignments of 92,283 additional potential Antarctic BLA sequences detected at the optimal thresholds for HMMER and MMSeqs2 against the structurally curated candidate protein set, supplemented with all BLDB BLA sequences and 24 non-BLA proteins from the same superfamilies. Points show amino acid identity and target coverage and are colored by protein class.

**Figure S3. Conservation of catalytic and other relevant motifs in divergent Antarctic class A beta-lactamases.** Multiple amino acid sequence alignment of key conserved regions among previously described class A beta-lactamases (shaded in green) and predicted Antarctic orthologs sharing 40-50% amino acid identity with the reference sequences. Conserved catalytic residues and characteristic class A beta-lactamase motifs remain preserved despite the substantial sequence divergence. PBP-5 (shaded in yellow) was included for comparison, as it belongs to the PBP-like superfamily but lacks beta-lactamase activity and was used as a true negative.

**Figure S3. Continued.**

**Figure S3. Continued.**

**Figure S3. Continued.**

**Figure S4. Conservation of catalytic and other relevant motifs in divergent Antarctic class C beta-lactamases.** Multiple amino acid sequence alignment of key conserved regions among previously described class C beta-lactamases (shaded in green) and predicted Antarctic orthologs sharing 50-60% amino acid identity with the reference sequences. Conserved class C catalytic motifs are preserved across the predicted Antarctic orthologs. AmpH protein (shaded in yellow) was included for comparison, as it belongs to the PBP-like superfamily but lacks beta-lactamase activity and was used as a true negative.

**Figure S5. Conservation of catalytic and other relevant motifs in divergent Antarctic class D beta-lactamases.** Multiple amino acid sequence alignment of key conserved regions among previously described class D beta-lactamases (shaded in green) and predicted Antarctic orthologs sharing 44-50% amino acid identity with the reference sequences. Conserved class D catalytic motifs and active-site residues are preserved across the predicted Antarctic orthologs despite their substantial sequence divergence. PBP2X protein (shaded in yellow) was included for comparison, as it belongs to the PBP-like superfamily but lacks beta-lactamase activity and was used as a true negative.

**Figure S5. Continued.**

**Figure S5. Continued.**

**Figure S5. Continued.**

**Figure S6. Conservation of catalytic and other relevant motifs in divergent Antarctic subclass B1 metallo-beta-lactamases.** Multiple amino acid sequence alignment of key conserved regions among previously described subclass B1 beta-lactamases (shaded in green) and predicted Antarctic orthologs sharing 47-50% amino acid identity with the reference sequences. Conserved residues and motifs relevant to subclass B1 metallo-beta-lactamases are largely preserved across the predicted Antarctic orthologs despite their sequence divergence. Chd protein (shaded in yellow) was included for comparison, as it belongs to the MBL-like superfamily but lacks beta-lactamase activity and was used as a true negative.

**Figure S6. Continued.**

**Figure S7. Conservation of catalytic and other relevant motifs in divergent Antarctic subclass B3 metallo-beta-lactamases.** Multiple amino acid sequence alignment of key conserved regions among previously described subclass B3 beta-lactamases (shaded in green) and predicted Antarctic orthologs sharing 40-50% amino acid identity with the reference sequences. Conserved residues and motifs relevant to subclass B3 metallo-beta-lactamases are largely preserved across the predicted Antarctic orthologs despite their sequence divergence. GloB protein (shaded in yellow) was included for comparison, as it belongs to the MBL-like superfamily but lacks beta-lactamase activity and was used as a true negative.

**Figure S7. Continued.**

**Figure S8. Functional similarity clustering of Antarctic candidate beta-lactamases and related true-negative proteins.** Uniform Manifold Approximation and Projection (UMAP) of functional similarities among reference beta-lactamases, Antarctic candidate beta-lactamases, and true-negative proteins. Each protein was represented as a 6,109-dimensional Gene Ontology term probability vector, and cosine distances between vectors were used for projection into two-dimensional space. Proteins are colored according to the protein class assigned from the reference proteins contained in each structural cluster.

**Figure S9. Regional normalized abundance of PBP-like beta-lactamases across Antarctic soil ecotypes.** Maximum-likelihood phylogenetic tree of representative Antarctic PBP-like beta-lactamases identified in this study. Colored bars indicate mean normalized abundance (copies per cell x 1,000) in each ecotype: Cold Desert (CD), Antarctic Peninsula (AP), and Subantarctic Islands (SI). Taxonomic affiliation and beta-lactamase class are shown alongside the corresponding branches. The figure highlights ecotype-specific abundance patterns that are not apparent when pooled abundance values are considered, revealing distinct biogeographic distributions of several class A, C, and D beta-lactamase lineages.

**Figure S10. Regional normalized abundance of MBL-fold beta-lactamases across Antarctic soil ecotypes.** Maximum-likelihood phylogenetic tree of representative Antarctic MBL-fold beta-lactamases identified in this study. Colored bars indicate mean normalized abundance (copies per cell x 1,000) in each ecotype: Cold Desert (CD), Antarctic Peninsula (AP), and Subantarctic Islands (SI). Taxonomic affiliation and beta-lactamase subclass are shown alongside the corresponding branches. The figure reveals ecotype-associated abundance patterns among subclass B1, B2, and B3 beta-lactamases, highlighting the heterogeneous biogeographic distribution of MBL-fold lineages across Antarctic and Subantarctic soils.

**Figure S11. Distribution of beta-lactamase classes across assembled metagenomic contigs and metagenome-assembled genomes.** Number of beta-lactamase-containing assembled metagenomic contigs (A) and metagenome-assembled genomes (MAGs) (B) recovered from Antarctic and Subantarctic soils. Bars are grouped according to beta-lactamase class and taxonomic affiliation, illustrating the phylogenetic distribution of the identified beta-lactamases across the microbial communities represented in the assembled datasets. This analysis highlights the broad taxonomic diversity of BLA-containing contigs and MAGs recovered from Antarctic soil microbiomes.

**Figure S12. Genomic contexts of Antarctic beta-lactamases associated with putative mobile genetic elements.** Representative assembled metagenomic contigs containing beta-lactamase genes located within or near recognizable mobile genetic element signatures. The upper panel shows one class D OXA-205-like beta-lactamase embedded in a complete integron and four beta-lactamase genes associated with CALIN-type integrons, including OXA-1172-like, GES-like, IDC-like, and OXA-1089-like variants. The lower panel shows beta-lactamase genes located near insertion sequence elements, including NOD-1-like, LRA10-1-like/class C, and CVI-1-like/subclass B2 beta-lactamases. Gene arrows indicate predicted coding sequences and their orientation. Beta-lactamase genes are color-coded according to structural class. These associations represent a limited subset of all identified Antarctic beta-lactamases and should be interpreted cautiously, as short-read assembly fragmentation limits complete reconstruction of genomic contexts and the presence of MGE signatures does not demonstrate active horizontal transfer.
